# Techno‐Economic and Lifecycle Analysis of Green Colloidal Silver: Moving toward Scale‐Up

**DOI:** 10.1002/gch2.202500263

**Published:** 2025-08-19

**Authors:** Federico Trotta, Danielle Winning, Dea Bozhani, Seyedeh Fatemeh Mirpoor, Stella Lignou, Sameer Khalil Ghawi, Dimitris Charalampopoulos

**Affiliations:** ^1^ Metalchemy Limited 71‐75 Shelton Street London WC2H 9JQ UK; ^2^ Department of Food and Nutritional Sciences University of Reading P.O. Box 226, Whiteknights Reading RG6 6AP UK

**Keywords:** advanced materials, colloidal silver, green synthesis, life‐cycle analysis, sustainable antimicrobials, techno‐economic analysis

## Abstract

Silver particles (AgPs) are increasingly used across a range of industries, including personal care, household, and food packaging, but conventional synthesis methods involve high production costs and negative environmental impacts. Green synthesis using plant extracts offers a sustainable alternative, though limited comparative data on economic and environmental performance exist. This study evaluates three green methods—BX3 (a patented extract), lemon juice (LJ), and green tea (GT)—against a conventional method using sodium borohydride (NaBH₄). Equal‐volume reactions are analyzed via ICP‐MS, UV–vis spectroscopy, and dynamic light scattering. Techno‐economic analysis and life cycle assessment (LCA) assessed costs and environmental impact. BX3 emerged as the most cost‐effective and environmentally friendly option, producing AgPs at $13,000/kg with a 75% yield and a global warming potential of 1,900 kg CO₂‐Eq/kg. In contrast, NaBH₄ yielded 7.35% at $195,000/kg, 15x more expensive than the BX3 method, and a global warming potential of 74,000 kg CO₂‐Eq/kg. GT, while a green method, has the highest cost $690,000/kg, the lowest yield (1.13%), and the worst environmental impact, including a human toxicity value of 92,000 kg 1,4‐DCB‐Eq/kg‐even surpassing the toxic NaBH₄ process. These findings highlight BX3's promise for scalable, low‐impact AgP production and broader industrial use.

## Graphical abstract

With increasing interest in colloidal silver for applications in healthcare, cosmetics, and pharmaceuticals, information on their synthesis methods and associated economic and environmental impacts remains limited. This study examines various green synthesis approaches, conducting a techno‐economic analysis and life‐cycle assessment, and compares them to conventional chemical synthesis to assess their implications for future colloidal silver production.

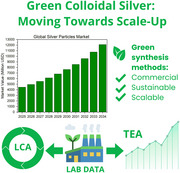

## 1 Introduction

Colloidal solutions have become a focal point of scientific and industrial research due to their distinctive physical, chemical, and biological properties, which differ significantly from their bulk counterparts.^[^ ^1^ ^]^ These unique characteristics largely stem from their high surface area‐to‐volume ratio, which enhances reactivity and interaction with other substances. Among various types of colloids, colloidal silver (CS) has attracted particular attention for its exceptional antimicrobial properties. This has led to a surge in their use across diverse sectors, including healthcare, food packaging, energy storage, and consumer products.

The growing demand for CS is closely linked to its proven effectiveness in inhibiting bacterial and fungal growth. They have demonstrated broad‐spectrum antimicrobial activity, with the ability to combat various different pathogens, and also reduce the unwanted toxicity to mammalian cells.^[^ ^2^ ^]^ As a result, AgPs are widely integrated into medical applications such as wound dressings, surgical tools, and implant coatings to reduce infection risks. In the food industry, they help extend shelf life by slowing microbial growth, while in cosmetics and household products, they offer preservative properties that align with rising consumer preferences for safer and more sustainable formulations.^[^ ^3^ ^]^

AgPs can be synthesized through three primary methods: physical, chemical, and biological. Physical methods, which involve a top‐down approach, use mechanical, electrical, or thermal forces to break down bulk silver into particles.^[^ ^4^ ^]^ Techniques such as grinding, laser ablation, or vapor condensation can yield high‐purity particles with good uniformity, but these approaches are typically energy‐intensive and less scalable. Chemical synthesis, a bottom‐up approach, is the most commonly used method in industry due to its relatively low energy requirements and efficient control over particle size and shape.^[^ ^5^ ^]^ This process involves the reduction of silver salts using chemical reducing agents. However, many of these agents—such as sodium borohydride, hydrazine, and dimethylformamide—are highly toxic and environmentally hazardous.^[^ ^6^ ^]^ This limits the application of chemically synthesized AgPs, especially in sensitive areas like food and healthcare.

As a response to these limitations, biological or “green” synthesis methods have gained attention for their potential to offer safer, more environmentally friendly alternatives. These methods use biological agents—such as bacteria, fungi, and plant extracts—to reduce silver ions into particles.^[^ ^7^ ^]^ Common plant‐based sources include lemon juice, green tea, and orange peels. Green synthesis is not only less toxic but may also reduce production costs by using readily available waste materials. However, despite its advantages, the economic and environmental viability of green synthesis remains insufficiently evaluated. Currently, there is a lack of comprehensive evidence demonstrating whether green methods are truly more sustainable than their chemical counterparts when scaled up for industrial production.

To effectively compare different silver particle synthesis routes, the use of techno‐economic analysis (TEA) and life cycle assessment (LCA) is crucial. These tools enable a comprehensive evaluation of both the economic and environmental dimensions of production processes. While LCA studies on different materials are becoming more common, many still suffer from limitations such as poor transparency and incomplete life cycle inventory (LCI) data—issues that are especially pronounced in the context of AgPs. Studies often assume ideal conditions, such as 100% yield, which do not reflect the realities of large‐scale manufacturing.^[^ ^8^ ^]^ Furthermore, few LCAs directly compare conventional and green synthesis routes, leaving a critical gap in our understanding of which methods offer the most sustainable path forward.^[^ ^5^ ^]^ This lack of reliable data and comparative insight hampers progress toward more responsible and scalable AgP production.

This study addresses these gaps by presenting a comparative TEA and LCA of green and conventional AgP synthesis methods. Specifically, it evaluates three green synthesis processes, using green tea extract (GT), lemon juice (LJ), and BX3, a proprietary plant‐based extract, against a widely used chemical synthesis route employing sodium borohydride (NaBH_4_). By assessing both the economic feasibility and environmental impact, this analysis aims to determine the most viable and sustainable method for producing AgPs at scale. The findings will help guide future decision‐making for investors, researchers, manufacturers, and policymakers interested in the sustainable development of colloids.

## 2 Results and Discussion

### 2.1 Macro and Micro Economic Trends of Silver Particle Production

The global interest in AgPs is not only driven by their scientific promise but also by shifting market dynamics, regulatory landscapes, and societal demands for safer and more sustainable technologies. As industries increasingly move away from harmful chemical agents traditionally used for preservation and antimicrobial functions, AgPs are emerging as a valuable alternative with significant economic and strategic potential.

Historically, many conventional antimicrobial agents, such as methylisothiazolinone (MIT), triclosan, and benzyl alcohol, have been extensively used in consumer goods, cosmetics, and industrial applications. However, growing awareness of their environmental and health risks has led to heightened regulatory scrutiny. These compounds are now facing bans or restrictions across multiple regions, particularly in the European Union and North America.^[^ ^9^, ^10^, ^11^ ^]^ Parallel to this trend, the global threat of antimicrobial resistance (AMR) has intensified the search for effective yet safer antimicrobial materials.^[^ ^12^ ^]^ As a result, there is growing interest in colloids solutions that include AgPs, which offer potent, broad‐spectrum antimicrobial activity with reduced potential for toxicity when used appropriately.

AgPs exhibit antimicrobial effects through the gradual release of silver ions, which disrupt cellular functions in microorganisms.^[^ ^13^, ^14^ ^]^ This sustained‐release mechanism allows for extended efficacy compared to traditional agents, making them especially attractive for applications requiring long‐lasting protection. However, despite these advantages, widespread adoption of AgPs has been limited by challenges related to scalability, consistency, and sustainability, particularly in the context of traditional chemical synthesis methods. These methods, such as those utilizing sodium borohydride as a reducing agent, are not only environmentally damaging but also difficult to implement at industrial scale due to safety concerns and process variability.^[^ ^15^, ^16^, ^17^ ^]^

One of the key limitations associated with conventional AgP production is the tendency for particles to aggregate during or after synthesis, particularly at larger volumes. This aggregation reduces the stability and antimicrobial efficacy of the particles, thereby limiting their practical utility. Moreover, batch‐based production methods often result in inconsistent reaction conditions, making it difficult to achieve uniform particle sizes.^[^ ^18^, ^19^, ^20^, ^21^ ^]^ Without standardized synthesis protocols and characterization techniques, comparing performance across different studies becomes problematic. These inconsistencies have also posed significant hurdles in the commercialization of AgPs, especially for applications that demand precise particle specifications and reproducible behavior.

**Figure 1:**
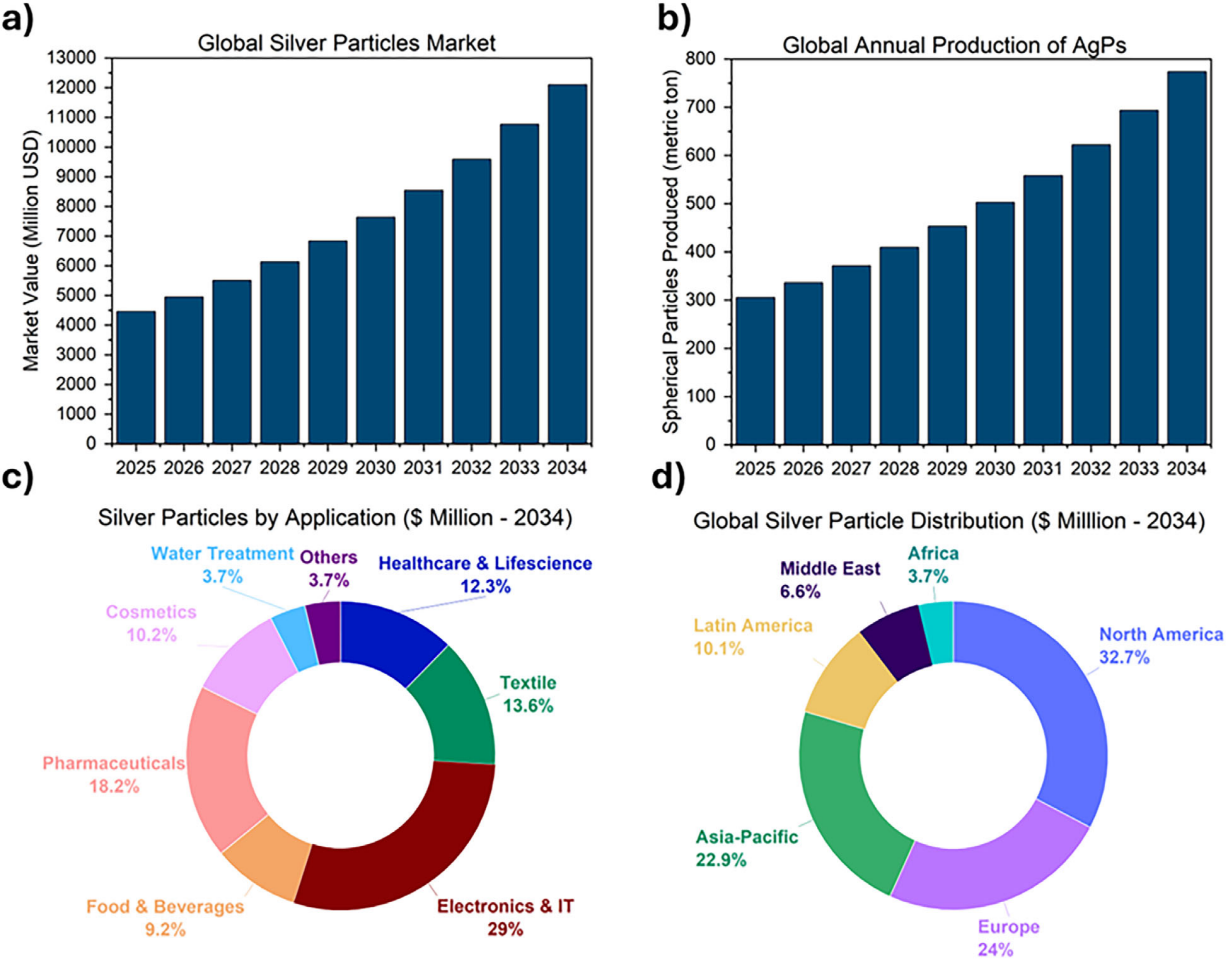
Global Market data of silver particles.

Despite these challenges, the global AgPs market has witnessed substantial growth in recent years. In 2024, the market was valued at ≈USD 4.02 billion and is expected to grow at a compound annual growth rate (CAGR) of 11.7% through 2034, as shown in **Figure** **1**a. By then, the market is projected to reach ≈USD 12.1 billion, underscoring its expanding role in advanced materials and healthcare sectors. The global annual production of spherical silver particles is projected to reach 774 tonnes by 2034, as shown in Figure 1b, growing at a CAGR of 10.9%, indicating strong and sustained market expansion driven by increasing demand across multiple high‐value applications.

Healthcare, in particular, has emerged as a dominant application area, accounting for roughly 35% of the total AgP market by 2023.^[^ ^22^ ^]^ This includes uses in wound care products, surgical implants, and medical devices, where infection prevention is paramount.^[^ ^23^ ^]^ For instance, Smith and Nephew, a leading medical technology firm, incorporates silver into its wound dressings to inhibit bacterial growth and accelerate healing.^[^ ^24^ ^]^ In the food industry, AgPs are increasingly used in antimicrobial packaging to extend shelf life and maintain product safety.^[^ ^25^, ^26^, ^27^ ^]^ Companies like Microban have developed technologies that integrate silver particles into packaging materials to prevent spoilage and contamination.^[^ ^28^ ^]^ Meanwhile, the energy sector is exploring AgPs for their role in enhancing the performance of batteries and solar energy devices, further expanding their commercial appeal.^[^ ^29^ ^]^ However, as shown in Figure 1c, electronics, IT, and pharmaceuticals are projected to be the leading application areas by 2034, highlighting the growing demand for particles suitable for these sectors.

Regionally, North America and Europe have historically led the global AgPs market, benefiting from strong R&D infrastructures, regulatory support, and well‐developed manufacturing capabilities.^[^ ^30^ ^]^ In 2024, North America accounted for nearly 31% of the global market revenue. Europe is projected to maintain steady growth at a CAGR of 11.7% through 2034, driven by increasing investments in technology and innovation‐driven sectors such as biotechnology and clean energy,^[^ ^22^ ^]^ as shown in Figure 1d. Meanwhile, the Asia‐Pacific region is rapidly emerging as the fastest‐growing market, with a projected CAGR of 12.1%. This growth is fueled by rising industrialization, expanding technological capabilities, and supportive government policies in countries like China and India.^[^ ^30^ ^]^

On the innovation front, AgP‐related research and development has been accelerating at a remarkable pace. Between 2007 and 2017, nearly 5,000 patent applications related to silver materials were filed.^[^ ^31^ ^]^ More recent data shows over 100,000 AgP‐related patents listed in international databases such as Google Patents and Espacenet, highlighting the growing intensity of innovation in this field.^[^ ^32^, ^33^ ^]^ Academic interest mirrors this trend, with over 10,000 peer‐reviewed articles on AgPs published between 2010 and 2020, covering advancements in synthesis techniques, performance optimization, and applications across various domains.^[^ ^34^ ^]^

Established companies play a crucial role in supplying AgPs for high‐impact sectors. For example, NanoComposix offers a wide range of metal and metal oxide nanoparticles tailored for use in nanomedicine, diagnostics, and electronics.^[^ ^35^ ^]^ Cytodiagnostics, based in Canada, produces AgPs for biomedical research and drug delivery, serving major institutions such as the National Institutes of Health (NIH), the University of Toronto, and pharmaceutical companies like Pfizer.^[^ ^36^ ^]^ Their 100 nm AgPs, at a concentration of 20 ppm, are priced at ≈$895 per kilogram.^[^ ^37^ ^]^ In comparison, Merck, another major player, sells similar particles for ≈$8,050 per kilogram, reflecting differences in branding, purity, and supply chain complexity.^[^ ^38^ ^]^ Notably, none of these companies currently offer green‐synthesized AgPs, pointing to a significant opportunity for innovation and market differentiation in this emerging segment.

Start‐ups are also increasingly entering the field, often backed by venture capital and government funding. Companies like Eascra Biotech are developing advanced DNA‐based nanostructures to enhance particle stability for biomedical applications, while Promethean Particles is scaling up the large‐volume production of inorganic nanoparticle dispersions. These companies have secured funding from major investors, including NASA, Aramco Ventures, and the British Business Bank, further validating the commercial potential of technology in this sector.

While these trends point to a promising future for AgPs, their successful commercialization hinges on compliance with regulatory frameworks that govern nanomaterials. In the UK and EU, nanomaterials are regulated under REACH (Registration, Evaluation, Authorisation and Restriction of Chemicals), while in the United States, the Toxic Substances Control Act (TSCA) applies. These regulations often depend on production volume thresholds and require detailed documentation regarding material composition, exposure risks, and safe handling practices.

Given the increasingly competitive and tightly regulated landscape, it is essential to rigorously evaluate both the environmental and economic performance of various AgP synthesis routes. Understanding the broader implications of these methods will be critical not only for commercial success but also for aligning with sustainability goals and meeting future regulatory expectations. A comprehensive comparison of green and conventional synthesis methods, therefore, offers valuable insights into which production pathways are most viable for responsible and large‐scale AgP manufacturing.

### 2.2 Silver Particles Synthesis and Characterisation

All green synthesis methods, along with the chemical synthesis using NaBH₄, successfully produced AgPs, as confirmed by the presence of characteristic surface plasmon resonance (SPR) bands in their UV–vis spectra (Figure S8, Supporting Information). **Table** **1** summarizes the yields and concentrations for each AgP suspension. Among the samples, BX3 exhibited the highest peak absorbance (22.7), followed by LJ at 5.55, with NaBH₄ (0.630) and GT (0.420) showing comparable but much lower values, as shown in Figure S8 (Supporting Information). These results align closely with the measured yields and masses, highlighting BX3 as the most efficient synthesis method, achieving a notably high yield of 74.5%.

**Table 1:** Summary of all key results for each of the AgP synthesis methods.

Characteristic	AgPs_BX3_	AgPs_LJ_	AgPs_GT_	AgPs_NaBH4_
Yield (%)	74.5	36.3	1.13	7.35
Post‐Filtration Concentration by ICP‐MS (µg mL^−1^)	461	107	15.8	9.80
AgP concentration by UV–vis (µg mL^−1^)	480	128	22.6	27.0

A consistent trend is observed in the pre‐ and post‐filtration concentrations measured by ICP‐MS for the LJ, GT, and NaBH₄ syntheses, where concentrations approximately halve after filtration. This decrease can likely be attributed to the larger hydrodynamic diameters measured for these samples. Larger particle sizes increase the likelihood of particle loss during filtration, which is reflected in the significant drop in post‐filtration ICP‐MS values.

### 2.3 Production Costs of Silver Particles

**Figure 2:**
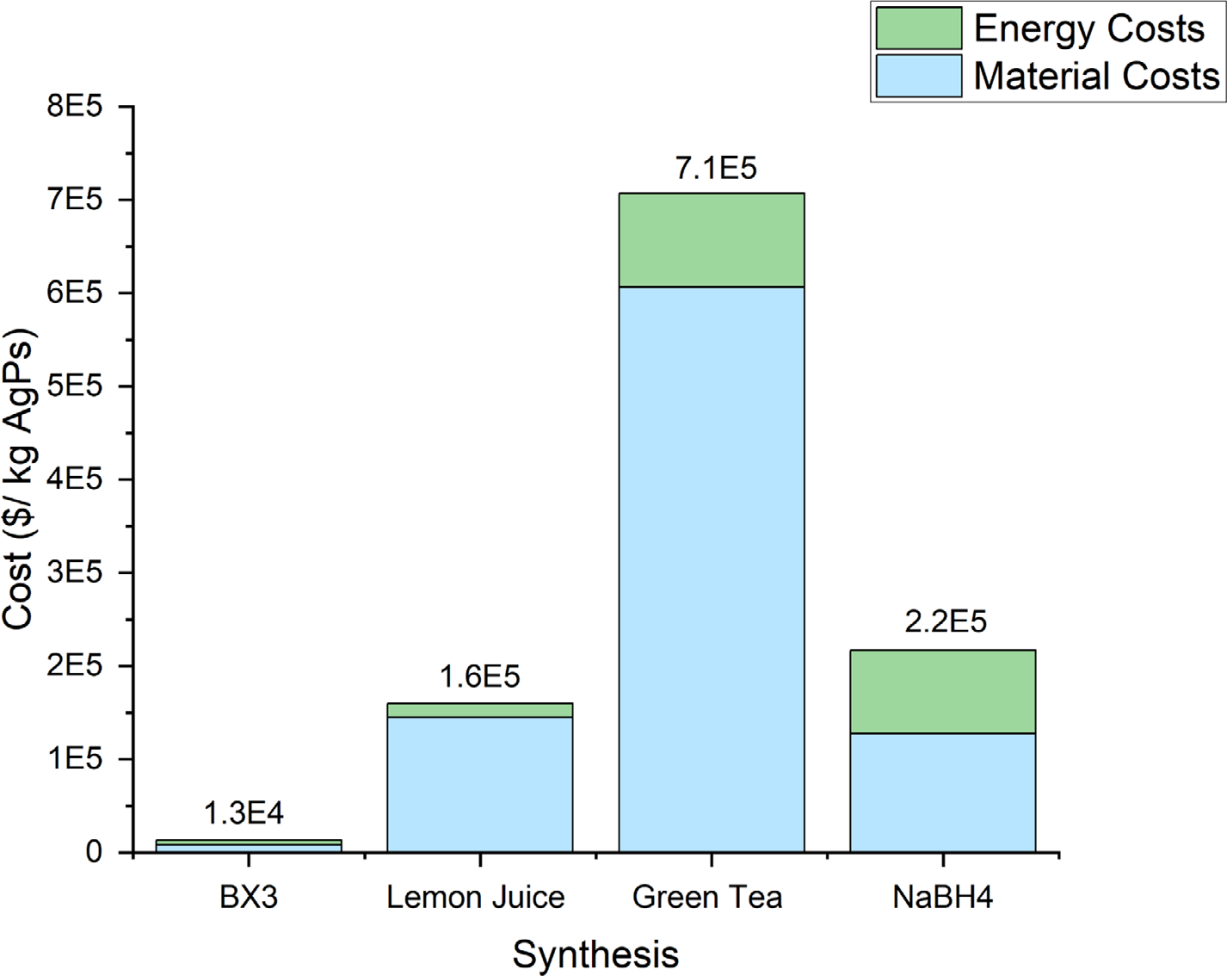
Total cost of silver particle (AgP) production for each synthesis method (BX3, Lemon Juice, Green Tea, and NaBH₄), expressed in USD per kilogram AgPs produced. Bars are stacked to show the relative contributions of material costs and energy costs to the overall production cost.

The production costs of 1 kg of AgPs were calculated (Table S9, Supporting Information), and the results are summarized in **Figure** **2**.

Figure 2 clearly illustrates that the synthesis using BX3 is significantly cheaper, with a total cost of $13,000 per kg of AgPs, compared to the LJ synthesis, which costs $160,000 per kg of AgPs, over 10 times more expensive than BX3. BX3 was found to be 50 times cheaper than GT, and nearly 15 times cheaper than the NaBH_4_ synthesis. This result aligns with the expectations and can be explained by the data in Table 1, where the BX3 synthesis produces a higher yield of AgPs within a 110 mL reaction volume compared to the other methods. However, the GT synthesis does not follow the expected cost trend; it remains more expensive than NaBH_4_ despite producing a higher quantity of AgPs. This discrepancy is due to the significantly higher amount of AgNO_3_ required (over 10 times more), which substantially increases the overall cost. In addition, GT is the least efficient in comparison to the other methods, only resulting in a yield of 1.13% as presented in Table 1.

**Figure 3:**
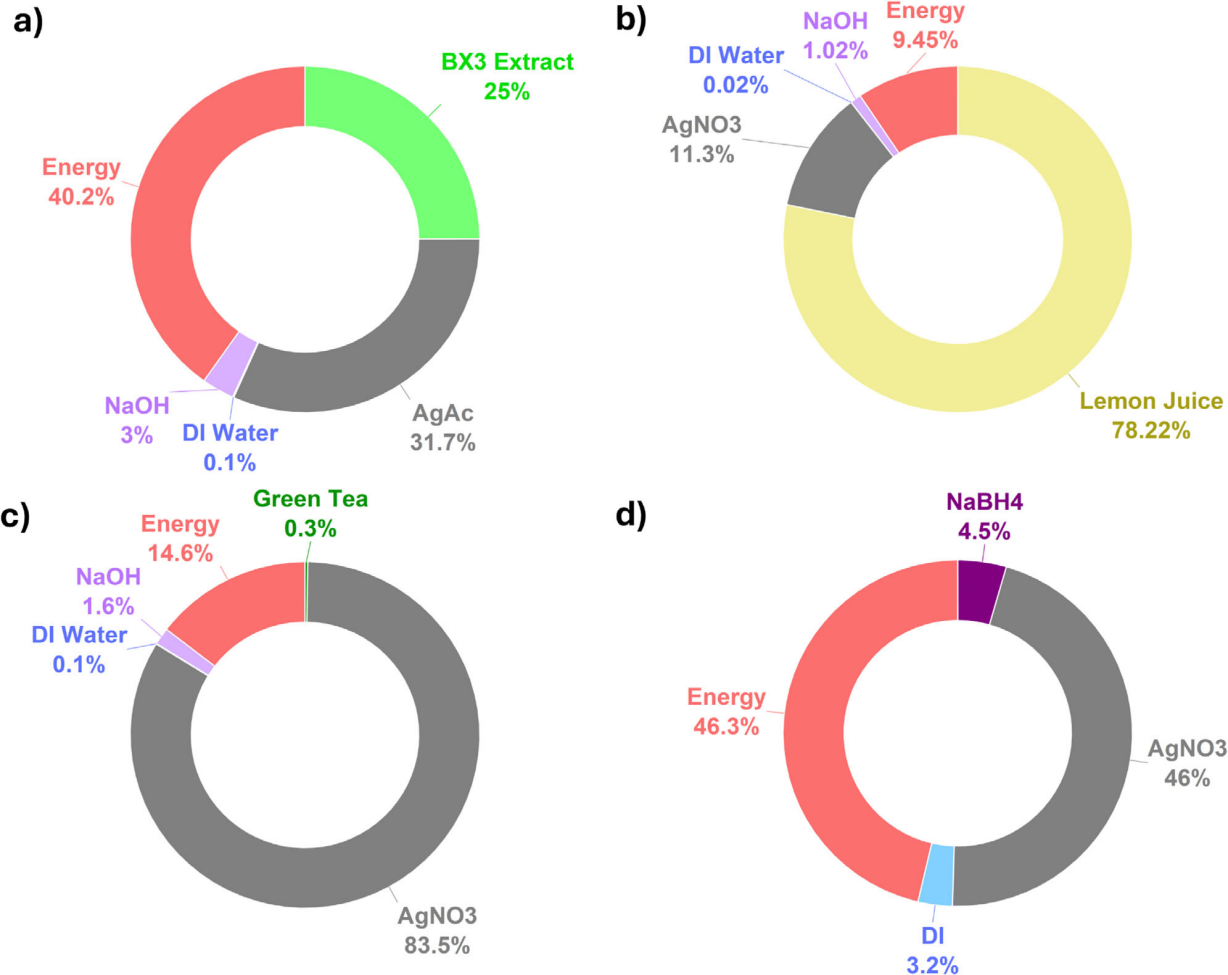
Cost breakdown of silver particle (AgP) syntheses using a) BX3, b) Lemon Juice, c) Green Tea, and d) NaBH₄ as reducing agents. Each pie chart displays the relative contributions of individual material costs and energy consumption to the total synthesis cost.

A more detailed cost breakdown (**Figure** **3**) for each synthesis provided further insights into these trends.

Energy presents the most cost‐intensive component of the BX3 synthesis, accounting for 40% of the total cost. This is expected due to the multiple steps involved, including the initial extraction of the BX3 solution followed by particle synthesis, as outlined in the process flow diagram (PFD) (Figure S1, Supporting Information). In contrast, the LJ synthesis only involves particle formation, where lemon juice itself constitutes the most significant cost contributor, comprising 78% of the total production cost. GT's cost is heavily affected by the cost of AgNO_3,_ which contributes to 84% of the total cost, as the synthesis requires a larger amount of the chemical. On the other hand, despite not requiring many steps for the synthesis, energy along with AgNO_3_ is still the most significant cost in the NaBH_4_ synthesis, attributing to 46% of the total cost each. This process requires less materials, and the synthesis itself is less energy intensive, however, it results in a low yield of AgPs.

### 2.4 Cost Optimization Strategies

Having assessed the costs of each synthesis, one way to reduce these costs and optimize the overall production cost is by minimizing the material costs. This can be achieved by buying from bulk suppliers like IndiaMart or Alibaba when carrying out the synthesis at a scale‐up.

**Table** **2** shows a significant decrease in the total cost for each synthesis in comparison to the initial costs observed in Figure 1. These changes are only due to the change in material costs. The cost of GT decreased by 74%, the most out of all the syntheses. This can be attributed to the fact that in Figure 3c, it can be seen that the cost of AgNO_3_ has the most impact for this synthesis, therefore, by decreasing this cost, it has a substantial impact on the overall cost of the process. On the other hand, for processes such as BX3 and NaBH_4,_ where energy was the most influential cost, a decrease in the cost of materials had an impact of 58% and 47% respectively, not as significant as that of GT. Nonetheless, the trend of the costs remains the same, with BX3 being the cheapest and GT the most expensive process to run per kg of AgPs produced. After optimizing material costs, energy expenses become the largest portion of the overall price. Therefore, this makes energy optimization a key area for further reducing overall costs.

**Table 2:** Original and optimized costs of silver particle (AgP) production for each synthesis method, expressed in USD per kilogram of AgPs. Cost optimization was achieved through reductions in material expenses, with the percentage change reflecting the cost decrease relative to the original value.

Synthesis	Original Cost [$/ kg AgPs]	Optimized Cost [$/ kg AgPs]	% Decrease
BX3	13,000	5,400	58%
LJ	160,000	55,000	65%
GT	690,000	180,000	74%
NaBH_4_	190,000	100,000	47%

In this study, synthesis was conducted in small batches using equipment designed for higher volume capacities. One effective strategy to reduce energy consumption is to process larger batches, thereby increasing throughput per unit of energy. Additionally, carrying out the syntheses in batch mode can lead to unreproducible size distributions and quality of particles produced.^[^ ^18^ ^]^ A continuous process leads to increased product selectivity, faster throughput, and higher product quality, which would result in a decrease in energy consumption and therefore costs.^[^ ^39^ ^]^

### 2.5 Process Critical Assessment

The current TEA provides an easy comparison between the processes, giving an overview of the production costs. However, it does not include labour and fixed costs such as facilities costs and overheads, which would impact the overall costs. In addition, the experiments were carried out in small laboratory‐scale environments of 110 mL batches, and in the case of scaling up the production, the costs would not linearly increase and might be significantly different. Also, costing has been calculated for environmental impact assessment and overall process efficiency benchmarking only and is not indicative of commercial pricing, as this may vary significantly depending on raw materials supply chain fluctuations, energy prices, and further operational costs beyond materials and energy. Moreover, shape, size, purity and end use application, and regulatory framework, as well as the final formulation composition, such as buffers and stabilisation agents, will significantly impact the final commercial costing and pricing.

Therefore, future analyses should account for all relevant cost factors and include experiments conducted in reactors at a larger scale, as well as sensitivity analysis on the different cost factors. In terms of cost optimization, this study focused on optimizing material costs and did not optimize the energy costs. Therefore, ways in which energy costs could be minimised should be explored and modelled.

### 2.6 Life‐Cycle Assessment of Silver Particles

The LCA calculations carried out for the transportation are found in s10 (Supporting Information). Herb origins for the calculation of the freight transport, in addition to the life cycle inventory for each of the synthesis processes, are summarized in Tables S11,S12 (Supporting Information), respectively.

#### 2.6.1 Global Warming Potential (GWP)

**Figure 4:**
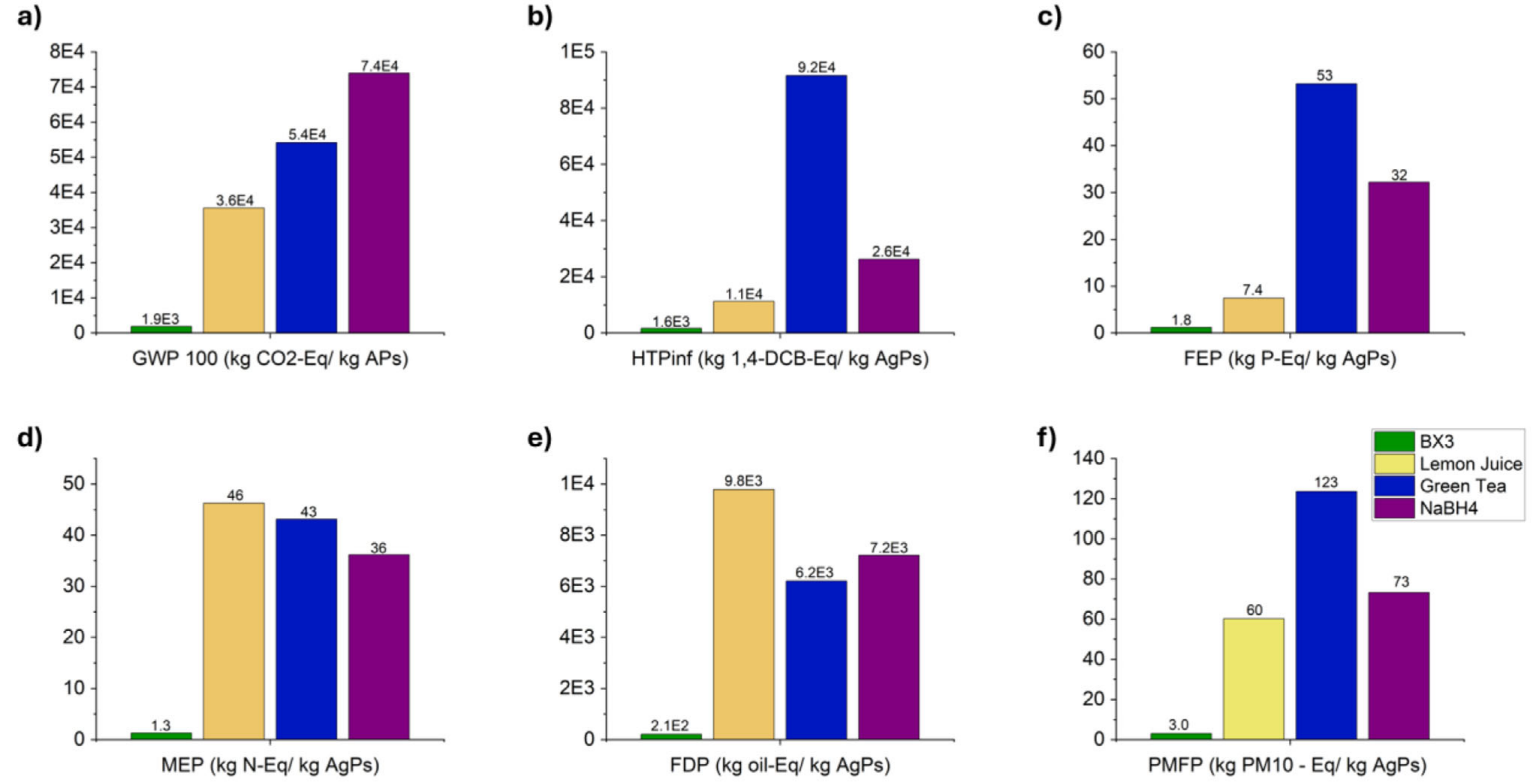
Life cycle assessment results for the synthesis methods using BX₃, lemon juice, green tea, and NaBH₄. The figure illustrates the environmental impacts across the following categories: a) Global Warming Potential over a 100‐year horizon, b) Human Toxicity Potential, c) Freshwater Eutrophication Potential, d) Marine Eutrophication Potential, e) Fossil Depletion Potential, and f) Particulate Matter Formation Potential.

The global warming potential 100 (GWP 100) evaluates the amount of greenhouse gases produced from a process over a 100 year time horizon and is measured as a CO_2_ equivalent. This allows companies to assess the impacts of a process on the environment and therefore develop strategies to lower them. As seen in **Figure** **4**a, the BX3 process contributes the lowest amount at 1,900 kg CO_2_–Eq per kg of AgPs produced. LJ and GT contribute 36,000 and 54,000 kg Co_2_‐Eq per kg of particles produced, respectively, while NaBH_4_ accounts for 74,000 kg Co_2_‐Eq per kg of AgPs – nearly 40 times larger than BX3, or ≈72,000 kg CO_2_–Eq per kg AgPs more. Demand of AgPs is consistently growing, and so applying BX3 as a green synthesis method would lead to a significant decrease in the environmental impacts and would expand the applications of AgPs within the mainstream industries, including consumer goods, shifting away from specialist niche applications.

Compared to the green methods, the chemical approach results in a greater negative impact per kilogram of particles produced. In the LCA carried out, the sodium borohydride used in this process was assumed to be synthesized via the Brown‐Schlesinger method, which is a complex industrial process consisting of 7 steps, leading to a higher environmental footprint.^[^ ^40^ ^]^ The production of sodium borohydride results in CO_2_ waste, which contributes to a high GWP as observed in Figure 4a.^[^ ^41^ ^]^

#### 2.6.2 Human Toxicity Potential (HTP)

The Human Toxicity Potential (HTP) quantifies the potential harm to human health from the release of toxic substances. This is measured as kg 1,4DCB equivalent, a suspected carcinogen. BX3 accounts for 1,600 kg 1,4DCB‐Eq per kg of AgPs, whilst GT contributes to 92,000 kg kg^−1^ AgP – nearly 58 times more than BX3 and ≈4 times higher than NaBH_4_, which stands at 26,000 kg 1,4‐DCB‐Eq per kg of AgPs (Figure 4b). Despite this process being a green method and using a natural reducing agent, the reaction mixture consists mainly of AgNO_3_. HTP considers the exposure to toxic substances, and in comparison to the other syntheses, GT requires nearly ten times more silver salt in the synthesis. For the 110 mL synthesis, GT requires 0.18 g of AgNO_3_ in comparison to LJ and NaBH₄, which require 0.037 and 0.017 g, respectively. Furthermore, the GT utilizes AgNO_3,_ which is considered more toxic and corrosive compared to silver acetate, which is used in the BX3 synthesis at a much lower quantity, i.e., only 0.077 g.^[^ ^42^ ^]^

#### 2.6.3 Freshwater and Marine Eutrophication Potential (FEP) (MEP)

Freshwater eutrophication (FEP), expressed in kg phosphorus equivalent (kg P eq), and marine eutrophication potential (MEP), expressed in kg nitrogen equivalent (kg N eq), assess the possible impacts of a process on freshwater and marine ecosystems. BX3 leads to 1.8 kg P‐Eq /kg of AgPs, whereas GT contributes the most, with 53 kg P‐Eq/kg AgPs, ≈30 times higher (Figure 4c). This can be primarily explained by GT's agricultural footprint, in addition to the production of more wastewater. Green tea production includes drying, which can be energy‐intensive. However, for MEP (Figure 4d), LJ, GT, and NaBH₄ display very similar values at 46, 43, and 36 kg N‐Eq, respectively, whilst BX3 remains the lowest and accounts for 1.3 kg N‐Eq. The slightly higher value for LJ can be attributed to the higher freight transport required, as well as the agricultural and processing emissions of lemons.

#### 2.6.4 Fossil Depletion Potential (FDP)

Fossil Depletion Potential (FDP) assesses the depletion of fossil fuels during a process and is measured in kg oil‐Eq per kg of AgPs produced. As observed in all of the graphs, for BX3, FDP is negligible in comparison to the other processes with a contribution of 210 kg in comparison to GT, which is the second lowest with 6,200 kg oil‐Equation (Figure 4e). LJ contributes the highest value to FDP among the compared processes, with a total of 9,800 kg oil‐Eq. This can be attributed to the significantly greater transport requirements for lemons, as they are assumed to be sourced from Spain. Additionally, the need for refrigeration during transit further increases their FDP. LJ can also be resource‐intensive in comparison to the other methods.

#### 2.6.5 Particle Matter Formation Potential (PMFP)

Particle Matter Formation (PMFP), measured as kg PM10 – Eq/ kg AgPs produced, refers to the formation of particulate matter in the atmosphere from certain substances and is a crucial factor in assessing the environmental impact of different processes.^[^ ^43^ ^]^ It is expressed as the mass of PM10 equivalent which are particles that are small enough to pass through the throat and nose and enter the lungs, leading to long‐term exposure such as reduced lung function and reduction in life expectancy.^[^ ^44^ ^]^ BX3 accounts for formations of 3 kg PM10 – Eq/ kg AgPs, the lowest value in comparison to LJ, NaBH_4,_ and GT, which contribute to 60, 73, and 123 kg PM10 – Eq/ kg, respectively (Figure 4f). GT leads to over 40 times more particulate matter formation in comparison to BX3. Particulate matter formation can be from combustion emissions, chemical emissions, transportation‐related pollution, and agricultural sources. GT's high value can be attributed to the use of pesticides and fertilizers, as well as drying, which all contribute to the production of particulate matter.

### 2.7 Overall Discussion

This study compared AgPs synthesized via green methods using BX3, LJ, and GT to a conventional chemical synthesis method using NaBH₄. Each synthesis was carried out at 110 mL batches for direct comparison of results. Through carrying out ICP‐MS at each stage of the methods, the mass balances of the whole process were modelled, and the final mass of AgPs was obtained. The BX3 process was the most efficient, with a 74.5% yield in comparison to GT, which had the lowest at 1.13%. Therefore, despite being the most energy‐intensive process due to the extra required steps for the preparation of the BX3 solution, when normalized per kg of AgPs produced, BX3 was significantly cheaper, at $13,000 per kg of AgPs produced. This is over 10 times cheaper than the second‐cheapest method, LJ.

Contrary to the other green methods which proved to be cheaper than the typical chemical method involving NaBH₄, the GT method was, in fact, the most expensive, which can be attributed to its extremely low yield.

A cost breakdown of each of the methods was modelled to gain insight into the primary factors impacting the overall cost. Whilst BX3 and NaBH₄ were primarily affected by the energy cost, LJ's most expensive factor was lemon juice, and GT's was AgNO_3_, attributed to the fact that it uses ≈10 times more than the other methods. Through this, a cost optimization analysis was carried out, and by considering material costs from a bulk buyer, the material costs significantly decreased, hence leading to a decrease in the overall cost. The cost of BX3 remained the cheapest at $5,400 per kg of AgP produced, however, the most significant impact was on GT with the highest percentage decrease of 74%. The study however, did not consider labour costs and fixed costs of equipment, which would change the calculated values. Additionally, scaling up the process does not result in a linear outcome, and transitioning from the current batch production to a continuous process could also impact energy costs. As such, this is another factor that warrants further investigation.

Through the implementation of an LCA, the impact of each of the methods on various factors was calculated and identified. BX3 proved to have the least environmental impact in comparison to the other synthesis methods, at times being over 20 times lower. This can be explained by the really high yield and so per kg of AgPs produced, the environmental impact is considerably less. This investigation clearly demonstrates that the green synthesis methods, BX3 and lemon juice, are significantly more cost‐effective than the conventional chemical approach using NaBH₄.

Among them, BX3 emerged as the most favorable overall, offering the best balance between economic viability and environmental sustainability.

## 3 Conclusion

In conclusion, AgPs synthesized using BX3 as the reducing agent demonstrated the most promising results for potential scale‐up and industrial implementation, offering both the lowest production costs and minimal environmental impact. Although green in nature, the synthesis using green tea performed the poorest overall, highlighting the need of LCA and economic analysis, even when compared to the conventional NaBH₄ method.

Future work should prioritize scaling up production and investigating the transition to continuous manufacturing. These steps would facilitate the integration of green synthesis methods into large‐scale processes and help accelerate the adoption of sustainably produced AgPs in industry.

## 4 Experimental Section

### Materials

Silver nitrate (AgNO_3_) (85229–50G), sodium hydroxide (NaOH) (S5881–500G), and silver(I) acetate (F494137–100G) were purchased from Fluorochem (UK). Sodium borohydride (NaBH_4_) (GPC9429W‐25G) was purchased from APC Pure (UK). Oregano, rosemary, watercress, kale, artichoke, lemon juice, and green tea bags were purchased from local supermarkets.

### Green‐AgPs Preparation

Each synthesis approach leveraged the diverse bioactive compounds present in natural extracts, which play a crucial role in both the reduction of silver ions (Ag⁺) to metallic silver (Ag⁰) and the stabilization of the resulting particles. Each synthesis was carried out at a starting volume of 110 mL.

### Green‐AgPs Preparation—Commercial Green Synthesis of AgPs (AgPs_BX3_)

AgPs prepared from the plant extracts were synthesized using a pre‐prepared BX3 solution with a plant extract mixture of oregano, kale, rosemary, artichoke, and watercress in a proprietary formulation as described in the original patent.^[^ ^6^ ^]^ For the synthesis, a 93.5 mL solution of silver acetate was heated under vigorous magnetic stirring. The BX3 solution was subsequently added to start the bioreduction reaction and achieve a final silver acetate concentration as outlined in the patent.^[^ ^6^ ^]^ The reaction resulted in a rapid colour change of the solution from dark brown to black, signaling reaction completion. The resulting solution was then cooled in an ice bath. AgPs were purified by means of centrifugation (6700 × g for 15 min) and re‐dispersed in sodium hydroxide (0.1 m). AgPs were further purified by means of vacuum microfiltration (3 µm, Whatman grade 44). A detailed outline of the BX3 preparation and synthesis procedure can be found in S1 (Supporting Information).

### Green‐AgPs Preparation—Lemon Juice Synthesis of AgPs (AgPs_LJ_)

AgPs were synthesised using an adapted method from Rajeshkumar et al.^[^ ^7^ ^]^ A 22 mL solution of AgNO_3_ (0.01 m) was added to commercially available lemon juice at a volume ratio of 1:4. The solution was subsequently heated to 80 °C and allowed to react for 10–15 min. The reaction was stopped when there was a colour change from colourless to brown. AgPs were isolated by means of centrifugation (6700 × g for 15 min) and re‐dispersed in sodium hydroxide (0.1 m). AgPs were separated from larger particulates by means of vacuum microfiltration (3 µm, Whatman grade 44). Information on details of the experiment can be found in S2 (Supporting Information).

### Green‐AgPs Preparation—Green Tea Synthesis of AgPs (AgPs_GT_)

AgPs were synthesised using an adapted method from Widatalla et al.^[^ ^45^ ^]^ The green tea extract was first prepared by heating dried green tea in deionised (DI) water (at 10 g L^−1^) to 60 °C. The extraction was carried out for 30 min under vigorous magnetic stirring. The solution was allowed to cool to room temperature and subsequently vacuum‐filtered (3 µm Whatman filter papers) to separate the extract. To prepare the AgPs, a 104 mL AgNO_3_ solution (0.01 m) was heated to 75 °C under vigorous stirring. The green tea extract was added dropwise until a colour change from colourless to yellow‐green to brown was observed. AgPs were isolated by means of centrifugation (6700 × g for 15 min) and re‐dispersed in sodium hydroxide (0.1 m). AgPs were separated from larger particulates by means of vacuum microfiltration (3 µm, Whatman grade 44). Detailed information on the reaction and exact measurements used for a 110 mL reaction mixture can be found in S3 (Supporting Information).

### Chemical‐AgPs Preparation

A common chemical synthesis reaction which uses sodium borohydride (NaBH_4_) was also performed to identify the differences between different synthesis methods and for comparison of results. It was common through literature for this method to use sodium borohydride as a reducing agent and then utilise Polyvinyl Alcohol (PVA) and Poly‐Vinyl‐Pyrrolidone (PVP) as stabilizing agents to prevent particle aggregation.^[^ ^46^ ^]^ However, in this study, NaBH_4_ was used as both the reducing and stabilizing agent.^[^ ^47^ ^]^

### Chemical‐AgPs Preparation—Sodium Borohydride Synthesis Method

AgPs were synthesized by first adding 10 mL 0.1 m AgNO_3_ to DI water under vigorous magnetic stirring. Ice‐cold NaBH_4_ solution (0.02 m) was added to the solution and stirred for 2–3 min. The change in colour from colourless to dark brown/black was almost immediate. AgPs were isolated by means of centrifugation (6700 × g for 15 min) and re‐dispersed in sodium hydroxide (0.1 m). AgPs were separated from larger particulates by means of vacuum microfiltration (3 µm, Whatman grade 44). The masses of each material used and reaction details can be found in S4 (Supporting Information).

### AgPs Characterisation—AgPs UV–vis Analysis

UV–vis absorption spectra of the AgPs were analysed in the wavelength range of 300–600 nm at room temperature (25 °C) with a Shimadzu UV‐1900i spectrophotometer (Tokyo, Japan). Samples were measured at a volume of 3 mL in disposable cuvettes (Scientific Laboratory Supplies, UK; BR759005). DI water was used as a reference. The concentration of each sample in µg mL^−1^ was determined using a calibration curve (Table 1^[^ ^48^ ^]^), which relates the peak absorbance to a concentration.

### AgPs Characterisation—Inductively Coupled Plasma Mass Spectrometry (ICP‐MS)

Silver standards across a concentration range of 1–1000 ppb were prepared using a serial dilution method in triplicate. In addition, standards for each AgP suspension (AgPs_BX3_, AgPs_LJ_, AgPs_GT_, and AgPs*NaBH*_4_) were prepared using a serial dilution method from the initial synthesised stock solution. To prepare the test samples, suspensions were first diluted using DI water to a maximum absorbance of 1 circa (corresponding to ≈20 µg mL^−1^). An aliquot (100 µL) of the diluted suspensions was further diluted using DI water by a factor of 1000 to achieve solutions within a ppb concentration range. All samples were analysed using a Thermo Scientific iCAP‐Q, run in Kinetic Energy Discrimination (KED) mode with a dwell time of 0.01s.

### Laboratory‐Scale Production Model and Techno‐Economic Evaluation

Microsoft Excel was used to model the mass balances of each experiment with a starting reaction mixture of 110 mL. Predefined values served as the inputs for the calculations, and the results from the experiments were used as outputs. ICP‐MS analysis was performed on the waste streams of each step to measure the concentration of silver ions and AgPs. For the ICP‐MS carried out for the supernatant, it was assumed that the solution consisted of 90% silver ions and 10% AgPs, whereas the ICP‐MS carried out on the samples of pre‐ and post‐filtering were assumed to be 90% AgPs and 10% silver ions. This allowed for the calculation of the total of AgPs and the reaction efficiency to be calculated.

The techno‐economic analysis was carried out by calculating the costs of each of the reactants per kg. This was then multiplied by the amount required for the 110 mL reaction solution and normalized to obtain the cost of producing 1 kg of AgPs. The time and electricity required for each step in one batch were recorded. The electricity consumption was measured by a smart plug sourced from KETOTEK. This calculated the exact amount of electricity units (kWh) consumed by the apparatus it was measuring and the time of use. This method was preferred over creating energy models for each step due to its increased accuracy. Using this data, the raw material and electricity costs for each method of silver particle production could be estimated using Equations (1) and (2). These equations were used to calculate the costs associated with the 110 mL experiments and do not account for scale‐up considerations. The full process diagrams of each synthesis method can be found in Figures S1–S4 (Supporting Information). Assumptions made for the analysis, in addition to the input and output of the mass balances, can be found in S5 (Supporting Information) and Table S6 (Supporting Information), respectively.

Material Costs ($/*kg* *AgPs*):

(1) $$\frac{\mathrm{Cost}\;\mathrm{of}\;\mathrm{Raw}\;\mathrm{Material}\;\left(\$/\mathrm{kg}\right)\;*\;\mathrm{Mass}\;\mathrm{of}\;\mathrm{Raw}\;\mathrm{Material}\;\mathrm{Required}\;\mathrm{for}\;110\;\mathrm{mL}\;\mathrm{batch}\;\left(\mathrm{kg}\right)}{\mathrm{Mass}\;\mathrm{of}\;\mathrm{Silver}\;\mathrm{particles}\;\mathrm{produced}\;\mathrm{from}\;110\;\mathrm{mL}\;\mathrm{batch}\;\left(\mathrm{kg}\right)}$$

Energy Costs ($/*kg* *AgPs*):

(2) $$\frac{\mathrm{Energy}\;\mathrm{Cost}\;\left(\$/\mathrm{kg}\right)\;*\;\mathrm{Energy}\;\mathrm{Usage}\;\left(\mathrm{kWh}\right)}{\mathrm{Mass}\;\mathrm{of}\;\mathrm{Silver}\;\mathrm{particles}\;\mathrm{produced}\;\mathrm{from}\;110\;\mathrm{mL}\;\mathrm{batch}\;\left(\mathrm{kg}\right)}$$

Yield (%):

(3) $$\frac{\mathrm{Mass}\;\mathrm{of}\;\mathrm{silver}\;\mathrm{particles}\;\mathrm{produced}\;\left(g\right)}{\mathrm{Moles}\;\mathrm{of}\;\mathrm{Silver}\;\mathrm{Salt}\;\mathrm{used}\;\mathrm{in}\;\mathrm{the}\;\mathrm{experiment}\;\left(\mathrm{mol}\right)\;*\;\mathrm{Molar}\;\mathrm{mass}\;\mathrm{of}\;\mathrm{Silver}\;\mathrm{particles}\;\left(g/\mathrm{mol}\right)}*100\%$$

The cost was subsequently optimized by evaluating various bulk suppliers. The same procedure was repeated, using Equation 1 to calculate material costs, thereby yielding values for the optimized material expenditures.

### Life‐Cycle Assessment Methodology

A cradle‐to‐gate life cycle analysis was conducted on all four synthesis methods, taking into account both the direct emissions from the production of AgPs and the indirect impacts associated with resource extraction, agricultural cultivation, and energy consumption. For green tea and lemons, the database provides information on inputs such as seedlings, fertilizers, and pesticides, along with their packaging, which were included in the agricultural cultivation phase of the assessment. The results from each synthesis were normalized to get the LCA data for the production of 1 kg of AgPs. The study was conducted using the EcoInvent 3.7 database through OpenLCA 2.4.0 software. For the LCA, ReCiPe was the method utilised, with a midpoint indicator focusing on single environmental impacts. Insight into database information and assumptions made can be found in S7 (Supporting Information).

## Conflict of Interest

The funders had no role in the design of the study; in the collection, analyses, or interpretation of data; in the writing of the manuscript; or in the decision to publish the results. Federico Trotta, Danielle Winning and Dea Bozhani are employees of Metalchemy Limited.

## Supporting information



Supporting Information

Supporting Information

## Data Availability

The data that support the findings of this study are available in the supplementary material of this article.

## References

[1] S. Husain , A. Nandi , F. Z. Simnani , U. Saha , A. Ghosh , A. Sinha , A. Sahay , S. K. Samal , P. K. Panda , S. K. Verma , J. Funct. Biomater. 2023, 14, 47. 36662094 10.3390/jfb14010047 PMC9863943

[2] P. R. More , S. Pandit , A. D. Filippis , G. Franci , I. Mijakovic , M. Galdiero , Microorganisms 2023, 11, 369. 36838334 10.3390/microorganisms11020369 PMC9961011

[3] Maximize Market Research Pvt Ltd ., in Silver Nanoparticles Market – Global Industry Analysis and Forecast (2024‐2030), Maximize Market Research Pvt Ltd, Pune, India 2020.

[4] R. Abbas , J. Luo , X. Qi , A. Naz , I. A. Khan , H. Liu , S. Yu , J. Wei , Nanomaterials 2024, 14, 1425. 39269087 10.3390/nano14171425 PMC11397261

[5] M. Slotte , G. Metha , R. Zevenhoven , Int J Energy Environ Eng 2015, 6, 233.

[6] F. Trotta , Composition and Method (accessed: November 2022) https://patents.google.com/patent/GB2598715B/en.

[7] S. Rajeshkumar , J. Nanomater. 2021, 2021, 1.

[8] N. U. M. Nizam , M. M. Hanafiah , K. S. Woon , Nanomaterials 2021, 11, 3324. 34947673 10.3390/nano11123324 PMC8708326

[9] H.‐N. Go , S.‐H. Lee , H.‐J. Cho , J.‐R. Ahn , M.‐J. Kang , S.‐Y. Lee , S.‐J. Hong , Sci. Rep. 2020, 10, 4099. 32139713 10.1038/s41598-020-60966-8 PMC7058054

[10] M.‐K. Song , J. Eun Park , S.‐H. Ryu , Y.‐W. Baek , Y.‐H. Kim , D. Im Kim , et al., Environ Int 2022, 170, 107643. 36403329 10.1016/j.envint.2022.107643

[11] M. Nowak‐Lange , K. Niedziałkowska , P. Bernat , K. Lisowska , Sci. Rep. 2022, 12, 19068. 36352006 10.1038/s41598-022-22981-9 PMC9645328

[12] M.d. A. Salam , M.d. Y. Al‐Amin , M. T. Salam , J. S. Pawar , N. Akhter , A. A. Rabaan , M. A. A. Alqumber , Healthcare (Basel) 2023, 11, 1946. 37444780 10.3390/healthcare11131946 PMC10340576

[13] R. B. Foldbjerg , P. L. Olesen , M. Hougaard , D. A. Dang , H. J. Hoffmann , H. Autrup , Toxicology Letters 2009, 190, 156. 19607894 10.1016/j.toxlet.2009.07.009

[14] J. Talapko , T. Matijević , M. Juzbašić , A. Antolović‐Požgain , I. Škrlec , cardiology and dermatology. Microorganisms. 2020, 8, 1400. 32932967 10.3390/microorganisms8091400 PMC7565656

[15] X.‐F. Zhang , Z.‐G. Liu , W. Shen , S. Gurunathan , Int. J. Mol. Sci. 2016, 17, 1534.27649147

[16] A. Almatroudi , Open Life Sci 2020, 15, 819. 33817269 10.1515/biol-2020-0094 PMC7747521

[17] N. P. U. Nguyen , N. T. Dang , L. Doan , T. T. H. Nguyen , Processes (Basel) 2023, 11, 2617.

[18] Y. Herdiana , N. Wathoni , S. Shamsuddin , M. Muchtaridi , OpenNano 2022, 7, 100048.

[19] Rita. Lab to market: Ensuring success in nanoparticles technology transfer. NETO Innovation. Retrieved from https://www.neto-innovation.com/post/lab-to-market-ensuring-success-in-nanoparticles-technology-transfer (accessed: April 2024).

[20] T. Bruna , F. Maldonado‐Bravo , P. Jara , N. Caro , Int. J. Mol. Sci. 2021, 22, 7202. 34281254 10.3390/ijms22137202 PMC8268496

[21] C. J. Taylor , A. Pomberger , K. C. Felton , R. Grainger , M. Barecka , T. W. Chamberlain , R. A. Bourne , C. N. Johnson , A. A. Lapkin , Chem. Rev. 2023, 123, 3089. 36820880 10.1021/acs.chemrev.2c00798 PMC10037254

[22] Silver Nanoparticles Market Size, Share & Trends Analysis Report By Application (Electronics & Electrical, Healthcare, Food & Beverages, Textiles, Others), By Region, And Segment Forecasts, 2024 –2030, https://www.grandviewresearch.com/industry‐analysis/silver‐nanoparticles‐market#:~:text=The%20global%20silver%20nanoparticles%20market,rising%20demand%20for%20antimicrobial%20applications (accessed: 2025).

[23] F. Eker , H. Duman , E. Akdaşçi , A. M. Witkowska , M. Bechelany , S. Karav , Nanomaterials 2024, 14, 1618.39452955

[24] Smith+Nephew Medical Devices and Advanced Wound Care, https://www.smith‐nephew.com/en‐gb (accessed: 2025).

[25] F. Trotta , S. Da Silva , A. Massironi , S. F. Mirpoor , S. Lignou , S. K. Ghawi , D. Charalampopoulos , Polymers (Basel) 2024, 16, 941. 38611199 10.3390/polym16070941 PMC11013251

[26] F. Trotta , S. Da Silva , A. Massironi , S. F. Mirpoor , S. Lignou , S. K. Ghawi , et al., Polymers (Basel) 2023, 15, 4243. 37959923 10.3390/polym15214243 PMC10650736

[27] S. Ediyilyam , B. George , S. S. Shankar , T. T. Dennis , S. Waclawek , M. Cerník , V. V. T. Padil , Polymers (Basel) 2021, 13, 1680. 34064040 10.3390/polym13111680 PMC8196760

[28] In: Microban [Internet]. #creator, https://www.microban.com/antimicrobial‐solutions/environments/food‐service (accessed: 2018).

[29] M. Z. A. Mahmud , V. N. Singh , J. Nanomater. 2023, 2023, 1.

[30] Silver Nanoparticles Market, https://www.imarcgroup.com/silver‐nanoparticles‐market (accessed: 2025).

[31] W. Sim , R. T. Barnard , M. A. T. Blaskovich , Z. M. Ziora , Antibiotics 2018, 7, 93. 30373130 10.3390/antibiotics7040093 PMC6315945

[32] https://patents.google.com/?q=(silver+nanoparticles)&num=25&oq=silver+nanoparticles (accessed: 2025).

[33] Espacenet, https://worldwide.espacenet.com/patent/search?q=silver%20nanoparticles (accessed: 2025).

[34] A. Naganthran , G. Verasoundarapandian , F. E. Khalid , M. J. Masarudin , A. Zulkharnain , N. M. Nawawi , M. Karim , C. A. Che Abdullah , S. A. Ahmad , Materials (Basel) 2022, 15, 427. 35057145 10.3390/ma15020427 PMC8779869

[35] nanoComposix. In: nanoComposix, https://nanocomposix.com/?gad_source=1&gbraid=0AAAAAD1EUbSyLKWXwHJTICZDwO3plr51n&gclid=Cj0KCQjwh_i_BhCzARIsANimeoGW3d226p‐Jvi2OVhgyG9_De5MmzkHBOvn0wXtfvJqNiX01ei‐w‐6waAiu9EALw_wcB (accessed: 2025).

[36] Cytodiagnostics Inc. In: Cytodiagnostics Inc [Internet]. https://www.cytodiagnostics.com/?srsltid=AfmBOoqIXQRm8s479xWCEQcdawbLu‐T1_AybfBr_IIIFF0OevkCdc1LP (accessed: 2025).

[37] 100 16 Apr 2025, nm Silver Nanoparticles. In: Cytodiagnostics Inc. https://www.cytodiagnostics.com/products/100nm‐silver‐nanoparticles?variant=31212671795274.

[38] Silver, dispersion https://www.sigmaaldrich.com/GB/en/product/aldrich/730777 (accessed: 2025).

[39] IChemE. Our Research Focus: Converting batch production to continuous processing https://www.thechemicalengineer.com/features/our‐research‐focus‐converting‐batch‐production‐to‐continuous‐processing/ (accessed: 2025).

[40] (PDF) The Concept about the Regeneration of Spent Borohydrides and Used Catalysts from Green Electricity, In: ResearchGate https://www.researchgate.net/publication/279278176_The_Concept_about_the_Regeneration_of_Spent_Borohydrides_and_Used_Catalysts_from_Green_Electricity (accessed: 2025).

[41] H. Larsson , Comparing Life Cycle Assessment of Li‐on batteries and fuel cells in chargers for small electronic applications, 2011 [cited 15]. Available: https://publications.lib.chalmers.se/records/fulltext/152970.pdf (accessed: 2025).

[42] J. Mertens , A. Alami , K. Arijs , Arch. Toxicol. 2023, 97, 1859. 37195448 10.1007/s00204-023-03511-6 PMC10256634

[43] Inhalable Particulate Matter and Health (PM2.5 and PM10) https://ww2.arb.ca.gov/resources/inhalable‐particulate‐matter‐and‐health (accessed: 2025).

[44] Particulate matter (PM10 and PM2.5). https://www.health.nsw.gov.au/environment/air/Pages/particulate‐matter.aspx (accessed: 2025).

[45] H. A. Widatalla , L. F. Yassin , A. A. Alrasheid , S. A. Rahman Ahmed , M. O. Widdatallah , S. H. Eltilib , A. A. Mohamed , Nanoscale Adv 2022, 4, 911. 36131825 10.1039/d1na00509j PMC9419201

[46] C. V. Restrepo , C. C. Villa , Environ Nanotechnol Monit Manag 2021, 15, 100428.

[47] U. T. Khatoon , A. Velidandi , G. V. S. Nageswara Rao , Mater. Chem. Phys. 2023, 294, 126997.

[48] Silver Nanoparticle Properties . Cytodiagnostics Inc [Internet]. [cited 8 May 2025]. Available: https://www.cytodiagnostics.com/pages/silver‐nanoparticle‐properties?srsltid=AfmBOooxiUh54iBLHoP4NML0EgVSV1eS50eENUYaJI5v2EpchSnFvrmo.

