# Supplementary material for: Techno‐Economic and Lifecycle Analysis of Green Colloidal Silver: Moving toward Scale‐Up
Source: Glob Chall. 2025 Aug 19;9(9):e00263. doi: 10.1002/gch2.202500263 (PMC12418338; doi:10.1002/gch2.202500263)
Supplement: Supplementary file 1 — Supporting Information [file GCH2-9-e00263-s001.pdf]

Global
Challenges
Open Access

Supporting Information

for *Global Challenges.*, DOI 10.1002/gch2.202500263

Techno-Economic and Lifecycle Analysis of Green Colloidal Silver: Moving toward Scale-Up

Federico Trotta, Danielle Winning, Dea Bozhani, Seyedeh Fatemeh Mirpoor, Stella Lignou, Sameer Khalil Ghawi and Dimitris Charalampopoulos*

Supporting Information

Techno-Economic & Lifecycle Analysis of Green Colloidal Silver: Moving Towards Scale-Up

Federico Trotta[1,*], Danielle Winning[1], Dea Bozhani [1],Seyedeh Fatemeh Mirpoor[2], Stella Lignou[2], Sameer Khalil Ghawi[2] and Dimitris Charalampopoulos[2]

[1]Metalchemy Limited, 71-75 Shelton Street, London WC2H 9JQ, UK
[2]Department of Food and Nutritional Sciences, University of Reading, P.O. Box 226, Whiteknights, Reading RG6 6AP, UK
[*]Author to whom correspondence should be addressed.

S1: Preparation of BX3 AgPs:

This green-synthesis method utilises a patented plant extract combination of different plants which act as the reducing agent for the synthesis of AgPs. The extract is a combination of artichoke, kale, oregano, rosemary, and watercress. The BX3 liquid mixture was prepared by combining dried oregano, rosemary, watercress, kale, and artichoke at an optimised ratio as found in the patent [1]. This dried blend was then mixed with deionized (DI) water at a proprietary concentration [1] and heated. The resulting solution was centrifuged with the supernatant collected. This was then vacuum filtered to obtain the final BX3 solution used for the synthesis. To synthesize the silver particles, a silver acetate solution was prepared and heated while stirring until fully dissolved. Then BX3 mixture was then added to start the bioreduction reaction. The reaction was continuously stirred until a colour change was observed. The resulting solution was cooled in an ice bath. The AgPs solution then underwent its first stage of separation where it was placed into the centrifuge. The supernatant was discarded consisting of DI water and majority silver and acetate ions. The particle pellets formed were resuspended in 0.1M Sodium Hydroxide (NaOH) and the mixture was sonicated to obtain a dispersed AgP solution. Finally the solution was purified via vacuum microfiltration, separating the AgPs from the larger particles. A detailed process can be found in the patent [1].

The reaction that takes place is as follows:

$$AgC_2H_3O_2 + BX3 \rightarrow AgPs \ + \ C_2H_3O_2^{\ -} + Ag^+ + BX3$$

Silver Acetate dissociates into Ag^+ and $C_2H_3O_2^{\ -}$ in solution. Metalchemy's patented BX3 reduces the Ag^+ ions into AgPs. Acetate ions ($C_2H_3O_2^{\ -}$) remain in the solution and some leftover Ag^+ ions may remain if the reduction is incomplete. The reaction resulted in a rapid colour change of the solution from dark brown to black, signalling reaction completion.

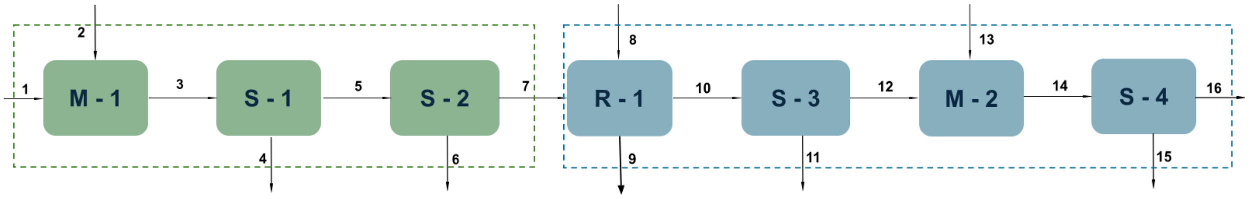

Figure S1. The process above depicts the green synthesis of Silver particles (AgPs) using a patented plant extract, BX3, as the reducing agent. The first three blocks (coloured in green) represent the production of the BX3 solution. M-1 is the mixing of the BX3 plant powders with water. S-1 is the centrifuge which is the first separation process of the process. S-2 is the filtration of the solution to produce the final BX3 solution used in the reaction. The next four blocks (coloured in blue) represent the synthesis of the AgPs where R-1 is the reaction block depicting the reaction of BX3 with silver acetate. S-3 is the separation of the AgP pellets using a centrifuge. M-2 represents the ultra sonicator. Finally, S-4 is the final filtration and yield of AgPs.

<u>S2: Preparation of Lemon Juice silver particles:</u>

22 mL of a solution of silver nitrate (0.01M) was added to 88 mL of commercially available lemon juice, creating a 1:4 volume ratio. The solution was subsequently heated to 80 °C and allowed to react until there was a colour change from yellow to dark brown. The reaction taking place is as follows:

$$AgNO_3 + LJ \rightarrow AgPs + NO_3^- + Ag^+ + LJ$$

The lemon juice acts as a reducing agent, reducing the Ag^+ ions into AgPs. The AgPs produced were isolated by means of centrifugation for 10 minutes at 6700 g-force, where the supernatant, consisting of DI water and majority silver and acetate ions, was discarded. The formed AgP pellets were re-dispersed in 40 mL of 0.1M Sodium Hydroxide (NaOH), then sonicated for 15 minutes to obtain a dispersed AgP solution. Vacuum microfiltration was then used to separate the AgPs from the larger particles.

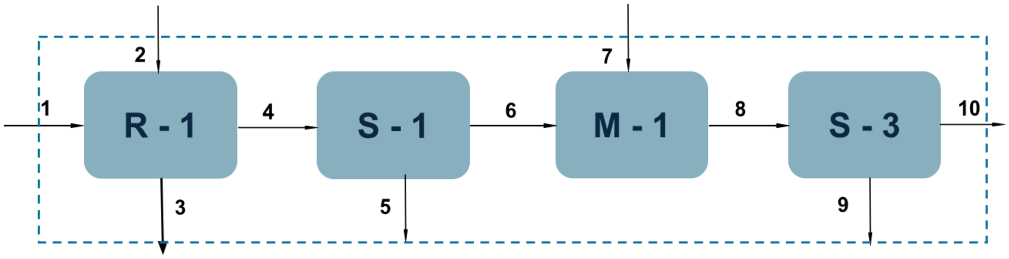

Figure S2. The process above depicts the green synthesis of Silver particles (AgPs) using Lemon Juice (LJ) as the reducing agent. The four blocks (coloured in blue) represent the synthesis of the AgPs where R-1 is the reaction block depicting the reaction Lemon Juice with silver nitrate. S-1 is the separation of the AgP pellets using a centrifuge. M-1 represents the ultra sonicator after the addition of 40 mL of NaOH to each test tube. Finally, S-3 is the final filtration and yield of AgPs.

S3: Preparation of Green Tea silver particles:

Green tea (GT) contains a high concentration of polyphenols, such as gallic acid, tannic acid, and epigallocatechin-3-gallate (EGCG) [2]. These compounds, due to their hydroxyl and carboxyl groups, can function as both reducing and stabilizing agents in the synthesis of silver particles. AgPs were synthesised using an adapted method from Widatalla et al. [3] For this synthesis method, 1 gram of dried GT samples, sourced from the Tetley company, was added to 100 ml of water. The mixture was heated for 30 minutes at 60°C under magnetic stirring, then allowed to cool and subsequently filtered using a vacuum filter. Meanwhile, a 104.6 mL solution of 0.01 M silver nitrate was heated to 70°C under magnetic stirring at 500 rpm. 5.4 mL of the green tea extract solution was then added dropwise from a burette into the heated silver nitrate solution, maintaining the magnetic stirring speed of 500 rpm. The formation of the silver particles was identifiable by a colour change from colourless to yellow to brown colour in the reaction mixture at which case the reaction was stopped. The reaction taking place is as follows:

$$AgNO_3 + GT \rightarrow AgPs + NO_3^- + Ag^+ + BX3$$

Silver nitrate dissociates into silver and nitrate ions, after which the GT acts as a reducing agent, converting silver ions into silver particles. The reaction mixture was then centrifuged for 10 minutes at 6700 g-force to separate the particles. The supernatant, primarily composed of deionized water, silver ions, and nitrate ions, was discarded. The remaining particle pellets were resuspended in 40 mL of 0.1M sodium hydride solution and sonicated for 15 minutes. Finally, the resulting dispersed AgP solution was filtered to separate the AgPs from larger particles.

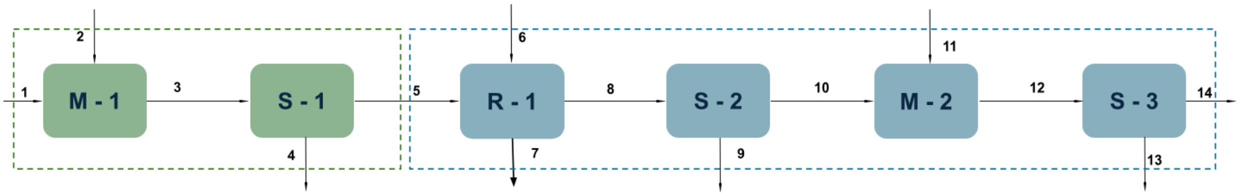

Figure S3. The process depicts the green synthesis of Silver particles (AgPs) using Green Tea (GT) as the reducing agent. The first 2 blocks (coloured in green) represent the production of the Green Tea solution. M-1 is the mixing and heating of the dried green tea leaves with DI water. S-1 is the filtration of the solution to produce the final GT solution. The next four blocks (coloured in blue) represent the synthesis of the AgPs where R-1 is the reaction block depicting the reaction of GT with silver nitrate. S-2 is the separation of the AgP pellets using a centrifuge. M-2 represents the ultra sonicator after the addition of 40 mL of NaOH to each vial. Finally, S-3 is the final filtration and yield of AgPs.

S4: Preparation of $NaBH_4$ silver particles:

Sodium borohydride ($NaBH_4$) is a commonly used reducing agent in the synthesis of silver particles. However, during the reaction, the side-product diborane decomposes in water, releasing hydrogen and forming boric acid. The reaction proceeds as follows:

$$2\,AgNO_3 + 2NaBH_4 + 6H_2O \rightarrow 2Ag + 2NaNO_3 + 2H_3BO_3 + 7H_2$$

10 mL of 0.01 M $AgNO_3$ was prepared by weighing 17 mg of $AgNO_3$ and dissolving in 10 mL of DI water. A 10 mL (0.02 M) solution of $NaBH_4$ was also prepared by dissolving 7.56 mg of $NaBH_4$ powder and dissolving in 10 mL of DI water and placed in the fridge. In the meantime, the 10 mL 0.01M $AgNO_3$ solution was added to 90 mL of DI Water under magnetic stirring at 500 rpm. Once the freshly prepared $NaBH_4$ reached ice-cold temperatures, this was added to the solution and stirred for 2-3 minutes. The change in colour from colourless to dark brown/black was almost immediate. The produced AgPs were separated via centrifugation at 6700 g-force for 10 minutes. The resulting pellets were kept whilst the supernatant, consisting of majority DI water, sodium nitrate and boric acid, was discarded. The AgP pellets were resuspended in 40 mL of DI water and sonicated for 15 minutes. Finally, the resulting dispersed AgP solution was filtered to separate the AgPs from larger particles

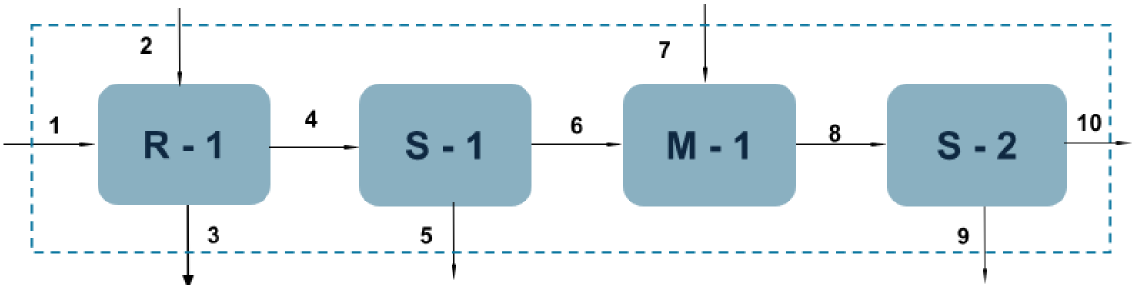

Figure S4. The process above depicts the green synthesis of Silver particles (AgPs) using sodium borohydride ($NaBH_4$) as the reducing agent. The four blocks (coloured in blue) represent the synthesis of the AgPs where R-1 is the reaction block depicting the reaction $NaBH_4$ with silver nitrate. S-1 is the separation of the AgP pellets using a centrifuge. M-1 represents the ultra sonicator after the addition of 40 mL of DI to each test tube. Finally, S-3 is the final filtration and yield of AgPs.

S5: Mass Balance Assumptions:

1. Two filter papers were used for the filtration of BX3 and GT.
2. It was assumed that all of the silver acetate and silver nitrate dissociates into silver ions and acetate/nitrate ions.
3. No water was used up in the reaction.
4. 90% of the supernatant was discarded with 10% remaining in the centrifuge tubes.
5. It was assumed that 90% of the supernatant concentration from the ICP-MS values was silver ions and 10% was AgPs.
6. The mass of acetate/nitrate ions was calculated from the moles of silver ions produced based on a 1:1 ratio, where the mass of silver ions was calculated from ICP-MS.
7. For the centrifuge, 2 centrifuge tubes were dilled to 50 mL, leaving 10 mL of waste to be discarded.
8. To calculate the mass of NaOH post filtration it was assumed that the NaOH retention is proportional to the AgP retention in the filter paper.

Table S6. Input and Output of reagents and products for all syntheses

	**$AgPs_{BX3}$ Process**	**$AgPs_{LJ}$ Process**	**$AgPs_{GT}$ Process**	**$AgPs_{NaBH_4}$ Process**
**Input (g)**				
Reducing Agent	1.3	88	0.062	0.0076
AgAC - BX3 reaction AgNO3 - other reactions	0.077	0.037	0.18	0.017
NaOH	0.32	0.32	0.32	0.0
DI	192	102	190	190
**Output (g)**				
Reducing Agent	1.49	7.95	0.53	0.00068
Silver Salt	0.0	0.0	0.0	0.0

DI Water	88	82	89	89
AgPs	0.037	0.0086	0.0013	0.00079
NaOH	0.31	0.16	0.14	0.0
Salt ions	0.0023	0.00055	0.000081	0.000051
Silver ions	0.0041	0.0010	0.00014	0.000088

S7: Life-Cycle Assessment Methodology:

As part of the analysis, this study examines the cultivation and production of green tea, lemon juice, oregano, rosemary, watercress, kale, and artichokes. For green tea and lemons, the database provides information on inputs such as seedlings, fertilizers, and pesticides, along with their packaging. Emissions from manure storage are excluded, as they fall within the system boundary of animal production. The production phase considers machine operations, including fertilizer and pesticide application, as well as irrigation. Additionally, direct field emissions and land use changes are accounted for. The energy required for drying green tea was sourced from literature on green tea production in China, a major supplier of green tea to the UK through Tetley [4]. The lemons were assumed to be from Spain as it is the dominant supplier of lemons in Europe [5].

The LCA database does not include specific data for the growth of oregano, rosemary, watercress, kale, and artichokes. Therefore, relevant inventories were sourced from literature. When certain information, such as the weight of packaging of fertilizers and pesticides, was missing, ratios similar to those used for green tea were applied. However, unlike in the production of green tea and lemons, trace amounts of metals and chemicals leaching into soil and water were not considered due to their minimal mass and lack of necessary data. This lack of reliable data is due to the variability on the amount of chemicals leached into the soil due to variability in soil types, weather conditions and farming practices, which could increase the uncertainty in the results.

A key decision was made to not utilise the Agribalyse 3.1 database due to the database utilising proxy data for the growth of the herbs and plants utilised in BX3. For example, it substitutes spinach data for watercress production, mint data for rosemary and oregano production whilst cauliflower data is utilised as a proxy dataset for artichoke and kale growth.

The sodium borohydride used in this process was assumed to be synthesized via the Brown-Schlesinger method, which proceeds as follows:

$$4NaH + B\left(OCH_3\right)_3 \rightarrow NaBH_4 + 3NaOCH_3$$

The data for the synthesis of sodium borohydride was provided by the EcoInvent 3.6 database.

LCA Assumptions:

1. The biobiowaste refers to the waste from the extraction of BX3 and GT.
2. Wastewater, unpolluted - refers to the waste from DI production and the value was assumed to be 80% of the tap water value input.
3. Wastewater, average - refers to supernatant waste from the centrifuge which contains silver and acetate ions as well as a small amount of AgPs.
4. Hazardous waste for incineration - refers to filter paper waste and test tubes for centrifugation.
5. NaBH4 includes waste products from the reaction:

$$2AgNO_3 + 2NABH_4 + 6H_2O -> 2Ag + 2NaNO_3 + 2H_3BO_3 + 7H_2$$

6. Mass of the side products were calculated using the mole ratio and from the mass of AgPs found from the mass balances.

Results and Discussion:

Uv-Vis graph:

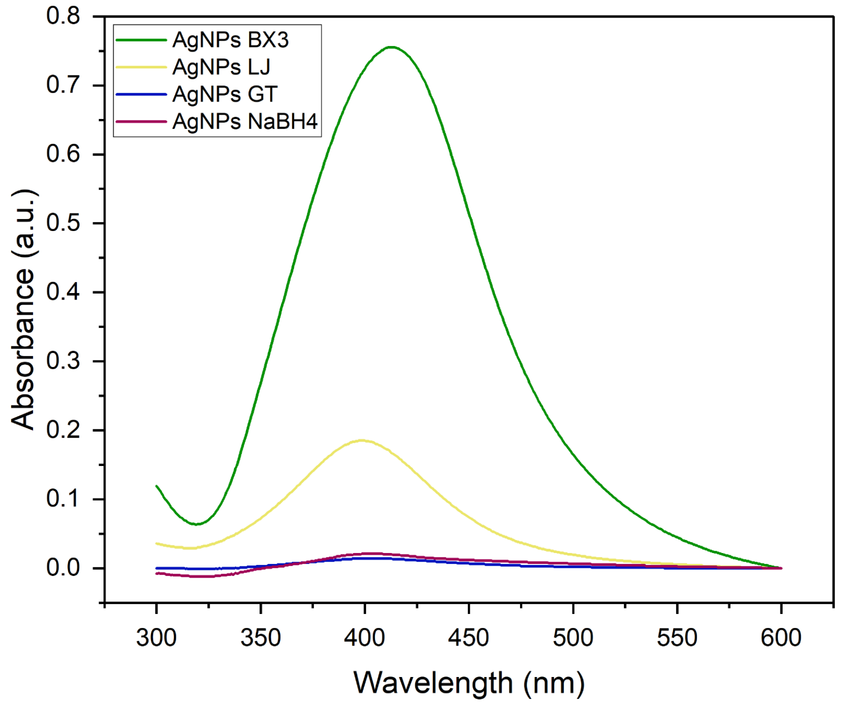

Figure S8. UV-Vis spectra of $AgPs_{BX3}$, $AgPs_{LJ}$, $AgPs_{GT}$, and $AgPs_{NaBH_4}$, recorded between 300 and 600 nm, showing characteristic SPR bands of AgPs.

Table S9. Material and Energy costs of each synthesis method per kg of silver particles produced.

Material	$/ $AgPs_{BX3}$	$/ $AgPs_{LJ}$	$/ $AgPs_{GT}$	$/ $AgPs_{NaBH_4}$
Reducing Agent	3090.45	124,000[6]	1770 [7]	8620 [8]
AgAc - for BX3 $AgNO_3$ for other syntheses	3929.86 [9]	17900 [10]	575000 [10]	88400 [10]
DI Water	13.23	30.3	383	6120
NaOH	374.69 [11]	1610 [11]	10900 [11]	-
Total material costs / kg AgPs	7,408.23	144,000	588000	103000
Process	Cost ($) $AgPs_{BX3}$	Cost ($) $AgPs_{LJ}$	Cost($) $AgPs_{GT}$	Cost ($) $AgPs_{NaBH_4}$
BX3/ GT Mixing	0.0173	-	0.0156	-
BX3 Centrifugation	0.0200	-	-	-
BX3/ GT Filtration	0.00124	-	0.00311	-
Reaction Synthesis	0.00219	0.0162	0.0121	0.0035
Centrifugation	0.0200	0.0200	0.0200	0.0200
Sonication	0.00829	0.00829	0.00829	0.0083
Filtration	0.0124	0.0059	0.00449	0.0045
Fume Hood	0.0983	0.0737	0.0590	0.0344
Water Filter	0.00484	0.00484	0.00484	0.0048
Total Energy Costs / kg AgPs	4976.37	15000	100000	89000

Energy costs were taken from electricity prices in the UK [12].

S10: LCA Transport calculations

For the Transportation calculations the distance was measured from the country of origin to the port in the UK and then truck distance from the port to the location of production.

Oregano and Rosemary:
Mersin (Turkey) → Southampton (England) → Location of Production (London)

Watercress and Kale:
Yantian (China) → London Gateway port → Location of Production (London)

Artichoke:
Hungary → Rotterdam (Netherlands) → London Gateway Port → Location of Production (London)

Lemon Juice:
Murcia (Spain) → Bilbao (Spain) → Liverpool (England) → Location of Production (London)

Green Tea:

Yantian (China) → London Gateway Port → Location of Production (London)

Table S11. Herb origin and freight transport calculations

Herb	Origin	Distance (Ship) (km)	kg*km	Distance (truck) (km)	kg*km
BX3 Extract	Turkey<br>China<br>Hungary	61653 [13–15]	22.8	2000	0.484
Lemons	Spain	1268 [16]	304	1139	273
Green Tea	China	24859 [14]	1.53	72	0.00443

Table S12. Life cycle inventory for each silver particle synthesis.

LCA requirements (g)	$AgPs_{BX3}$	$AgPs_{LJ}$	$AgPs_{GT}$	$AgPs_{NaBH4}$
Inputs				
Reducing Agent	1.3	88	0.06	0.01
AgAC - BX3 reaction AgNO3 - other reactions	0.077	0.037	0.177	0.017
NaOH	0.32	0.32	0.32	N/A
Tap Water	192.07	102	190	190
Ship Transport	22.8	304	1.53	N/A
Truck Transport	0.484	273	0.00444	N/A
Outputs				
AgPs	0.0317	0.0086	0.00127	0.0008
Colloidal Silver	88.0	89.5	88.9	89.5
Biowaste	3.37	N/A	0.28	N/A
Wastewater, unpolluted	154	81.6	152	152
Wastewater, Average	90.0	90.0	90.2	90.0
Hazardous Waste for incineration	23.1	22.2	23.1	22.2
1. Filter Paper	3.07	2.20	3.07	2.20
2. Test Tubes	20	20	20	20

References

1. GB2598715. [cited 8 May 2025]. Available: https://www.search-for-intellectual-property.service.gov.uk/GB2598715/documents

2. Singh BN, Shankar S, Srivastava RK. Green tea catechin, epigallocatechin-3-gallate (EGCG): mechanisms, perspectives and clinical applications. Biochem Pharmacol. 2011;82: 1807–1821. doi:10.1016/j.bcp.2011.07.093

3. Widatalla HA, Yassin LF, Alrasheid AA, Rahman Ahmed SA, Widdatallah MO, Eltilib SH, et al. Green synthesis of silver nanoparticles using green tea leaf extract, characterization and evaluation of antimicrobial activity. Nanoscale Adv. 2022;4: 911–915. doi:10.1039/d1na00509j

4. What is Tea? [cited 8 May 2025]. Available: https://www.tetley.co.uk/what-is-tea

5. European market potential for lemons. [cited 8 May 2025]. Available: https://www.cbi.eu/market-information/fresh-fruit-vegetables/lemons/market-potential

6. Sainsbury's online Grocery Shopping and Fresh Food Delivery. [cited 8 May 2025]. Available: https://www.sainsburys.co.uk/gol-ui/product/jif-lemon-lemon-juice-100ml

7. Sainsbury's online Grocery Shopping and Fresh Food Delivery. [cited 8 May 2025]. Available: https://www.sainsburys.co.uk/gol-ui/product/tetley-pure-green-tea-bags-x50

8. Sodium borohydride. [cited 8 May 2025]. Available: https://www.google.com/url?q=https://www.sigmaaldrich.com/GB/en/product/aldrich/452882&sa=D&source=editors&ust=1746737931320870&usg=AOvVaw3-g786r5DmIT_VErVnSGvm

9. Doug Discovery - the intermediate chemical compound catalogue - Fluorochem Ltd. [cited 8 May 2025]. Available: https://dougdiscovery.com/p/F494137

10. Doug Discovery - the intermediate chemical compound catalogue - Fluorochem Ltd. [cited 8 May 2025]. Available: https://dougdiscovery.com/p/F080282

11. Doug Discovery - the intermediate chemical compound catalogue - Fluorochem Ltd. [cited 8 May 2025]. Available: https://dougdiscovery.com/p/F996963

12. Yurday E. Average Cost of Electricity per kWh in the UK (2025). NimbleFins; 6 Jun 2018 [cited 8 May 2025]. Available: https://www.nimblefins.co.uk/average-cost-electricity-kwh-uk#:~:text=debit%20or%20prepayment.-,Electricity%20prices%20UK,2025%20through%2031%20June%202025.

13. Turkey to United Kingdom by Air freight, Cargo ship or Road - Fluent Cargo. [cited 17 Apr 2025]. Available: https://www.fluentcargo.com/routes/turkey/united-kingdom

14. China to United Kingdom by Air freight, Cargo ship or Road - Fluent Cargo. [cited 17 Apr 2025]. Available: https://www.fluentcargo.com/routes/china/united-kingdom

15. Hungary to United Kingdom by Air freight, Cargo ship or Road - Fluent Cargo. [cited 17 Apr 2025]. Available: https://www.fluentcargo.com/routes/hungary/united-kingdom

16. Spain to United Kingdom by Air freight, Cargo ship or Road - Fluent Cargo. [cited 17 Apr 2025]. Available: https://www.fluentcargo.com/routes/spain/united-kingdom
